# Assessment of a portable UV–Vis spectrophotometer's performance in remote areas: Stream water DOC, Fe content and spectral data

**DOI:** 10.1016/j.dib.2021.106747

**Published:** 2021-01-13

**Authors:** Xudan Zhu, Liang Chen, Jukka Pumpanen, Markku Keinänen, Hjalmar Laudon, Anne Ojala, Marjo Palviainen, Mikko Kiirikki, Kimmo Neitola, Frank Berninger

**Affiliations:** a Department of Environmental and Biological Sciences, University of Eastern Finland, 80101 Joensuu, Finland; b Department of Environmental and Biological Sciences, University of Eastern Finland, 70211 Kuopio, Finland; c Department of Forest Ecology and Management, Swedish University of Agricultural Sciences, 90183 Umeå, Sweden; d Faculty of Biological and Environmental Sciences, Ecosystems and Environment Research Programme, University of Helsinki, Niemenkatu 73, 15140 Lahti, Finland; e Institute for Atmospheric and Earth System Research/Forest Sciences, Faculty of Agriculture and Forestry, University of Helsinki, 00014 Helsinki, Finland; f Faculty of Biological and Environmental Sciences, Helsinki Institute of Sustainability Science, University of Helsinki, 00014 Helsinki, Finland; g Department of Forest Science, University of Helsinki, 00014 Helsinki, Finland; h Luode Consulting Sinimäentie 10 B, 02630 Espoo, Finland; i Institute for Atmospheric and Earth System Research (INAR), University of Helsinki, 00014 Helsinki, Finland

**Keywords:** Water quality, UV-Vis spectrophotometer, Spectral absorbance, Dissolved organic matter, Ferric iron

## Abstract

This paper presents data for the assessment of a portable UV-Vis spectrophotometer's performance on predicting stream water DOC and Fe content. The dataset contains DOC and Fe concentrations by laboratory methods, in-situ and ex-situ spectral absorbances, monitoring environmental indexes such as water depth, temperature, turbidity and voltage. The records in Yli-Nuortti river (Cold station, Finland) took place during the hydrological year 2018-2019 and in Krycklan (C4 and C5, Sweden) during the hydrological years 2016-2019. The data analyses were conducted with ‘*pls*’ and ‘*caret*’ package in R. The correlation coefficient (R), root-mean-square deviation (RMSD), standard deviation (STD) and bias were used to check the performance of the models. This dataset can be combined with datasets from other regions around the world to build more universal models. For discussion and more information of the dataset creation, please refer to the full-length article “Assessment of a portable UV–Vis spectrophotometer's performance for stream water DOC and Fe content monitoring in remote areas” [1].

## Specifications Table

**Table:** 

Subject	Environmental Science
Specific subject area	Water Science and Technology
Type of data	Table, Figure and Excel
How data were acquired	1. Portable multi-parameter UV–Vis probes (spectro::lyser, S::CAN Messtechnik GmbH, Austria) 2. UV-1800 UV-VIS spectrophotometer (Shimadzu, Kyoto, Japan) 3. Multi N/C 2100, Analytik Jena, Germany 4. Shimadzu TOC-5000 5. Victor3 1420 Multilabel Counter (PerkinElmer) 6. Inductively Coupled Plasma Optical Emission Spectroscopy (ICP-OES Varian Vista Pro Ax)
Data format	Raw and analysed.
Parameters for data collection	Water samples were collected from catchments in remote areas of the northern hemisphere with different degrees of water browning.
Description of data collection	The data collection in Yli-Nuortti river (Cold station) took place during the hydrological year 2018-2019 and in Krycklan (C4 and C5) during the hydrological years 2016-2019.
Data source location	Institution: Swedish University of Agricultural Sciences, University of Helsinki City/Town: Umeå / Krycklan, Lapland / Värriö Country: Sweden & Finland Location: 64˚14′ N, 19 ˚46′E, 67˚44′ N, 29 ˚27′E
Data accessibility	Repository name: Mendeley Data Identification number: https://doi.org/10.17632/f67dw4hccv.1 Direct URL to data: https://data.mendeley.com/datasets/f67dw4hccv/1[2]
Related research article	X. Zhu, L. Chen, J. Pumpanen, M. Keinänen, H. Laudon, A. Ojala, M. Palviainen, M. Kiirikki, K. Neitola, F. Berninger, Assessment of a portable UV–Vis spectrophotometer's performance for stream water DOC and Fe content monitoring in remote areas, Talanta. (2020) 121919. https://doi.org/10.1016/j.talanta.2020.121919[1]

## Value of the Data

• The data can be used to build accurate and unbiased models for multiple watersheds for DOC prediction in Northern Fennoscandia, and these models could be extrapolated from one watershed to another even without site-specific calibration for DOC.
• Scientific guidance could be provided to water industry and hydrological researchers for the applications of portable UV–Vis spectrophotometers for different purposes.
• When similar research in different regions around the world are conducted in the future, this data can be combined to prove the generality of the proposed models for DOC prediction.

## 1 Data Description

The development of continuously operating water quality sensors has led to a transition from studying long-term trends and seasonal patterns to the investigation of highly dynamic phenomena, such as storm events and diurnal patterns, using high-frequency in situ measurements [3]. With the currently available technology and decreasing costs, in situ sensors are more frequently used for monitoring, especially in remote areas [4], [5], [6]. Although large amounts of data present challenges regarding storage, processing, and analysis [7], long-term monitoring datasets provide an opportunity for detailed investigations of hydrological and biogeochemical processes in dynamic systems [6,8,9].

**Fig. 1:**
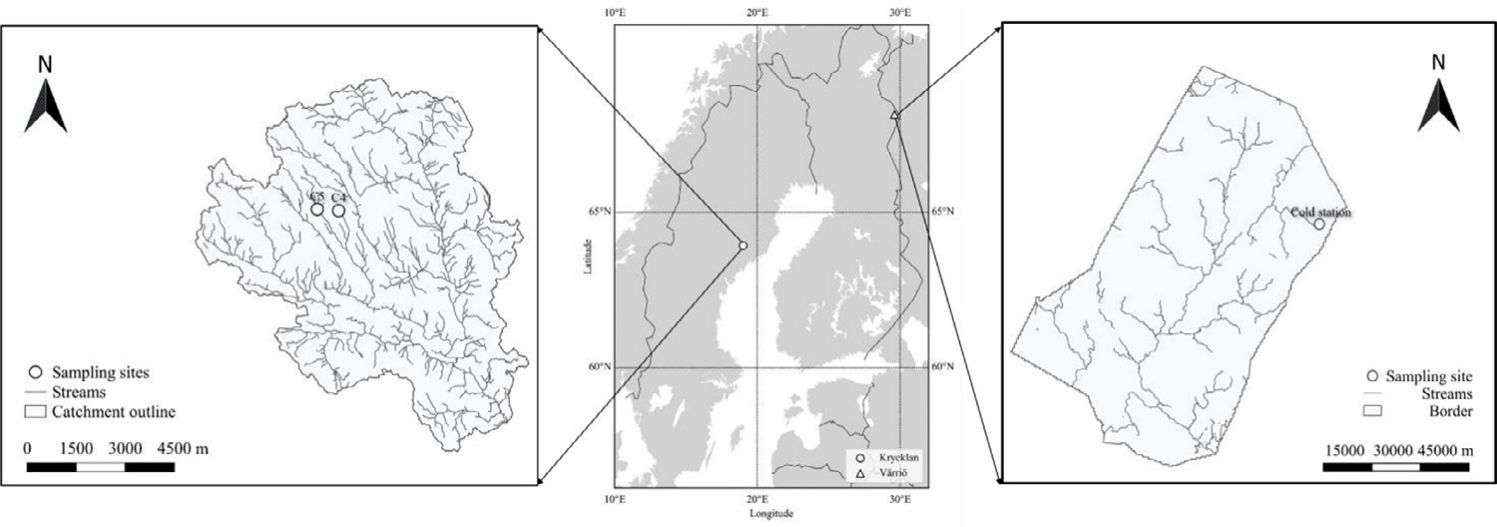
Site locations in Finland and Sweden.

This paper presents data for the assessment of a portable UV-Vis spectrophotometer's performance on predicting stream water DOC and Fe content in remote area. Fig. 1 is the site locations in Finland [2]in-situ and ex-situ spectral absorbance shows the performance of in-situ S::CAN (Fig. 3). The details of data sets for modelling are listed in Table 1. The performance of DOC and Fe predicted models are shown in Table 2-4 (for DOC) and Table 5-7 (for Fe), respectively. Raw data for each step of analysis are recorded in 4 excels which are available at the direct URL (https://data.mendeley.com/datasets/f67dw4hccv/1) [2]. Excel1 (In-situ & ex-situ absorbance) includes spectral absorbances measured by two methods (S::CAN and UV-1800) and their ratios. Monitoring environmental indexes such as water depth, temperature, turbidity, voltage and absorbance ratios in Cold station from 2018 to 2019 are listed in Excel2 (Absorbance & environmental index). Excel3 (Absorbance & lab measured DOC) contains in-situ daily spectral absorbances at wavelength 220 to 732.5 and laboratory measured DOC. Excel4 (Absorbance & lab measured Fe) indicates in-situ daily spectral absorbances at wavelength 220 to 732.5 and laboratory measured Fe.

**Fig. 2:**
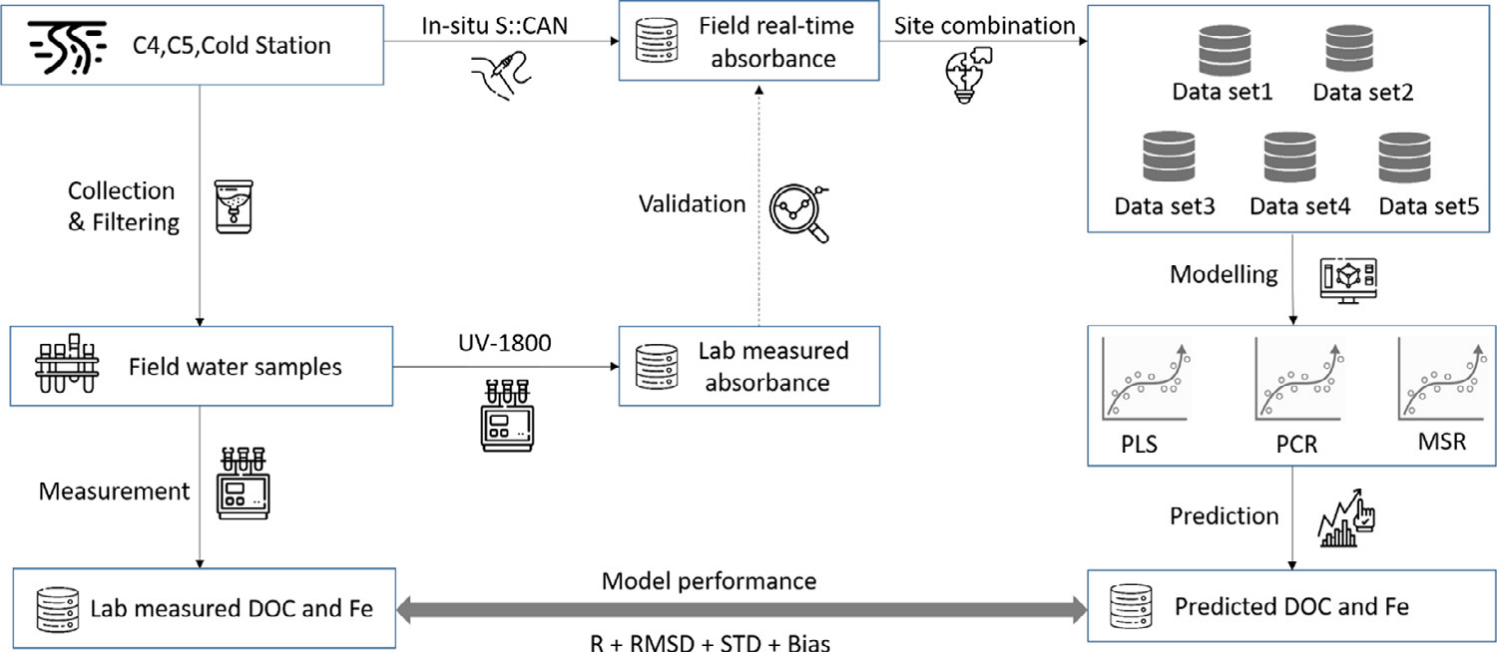
Experimental design.

**Fig. 3:**
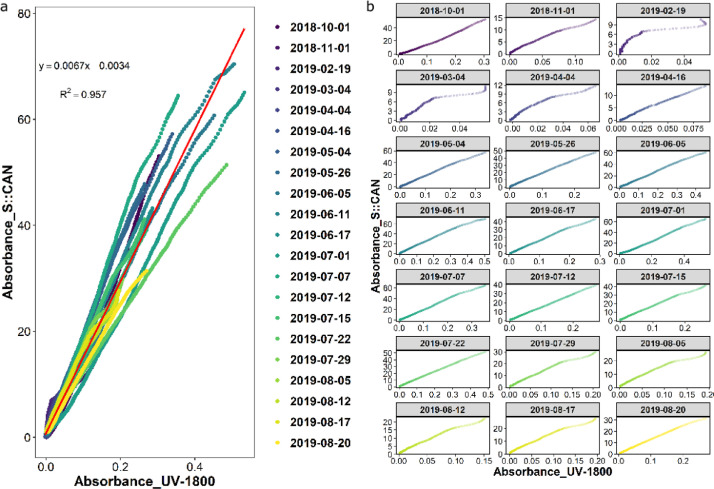
Relationship between Spectral absorbance measured by UV-1800 and S::CAN in different days.

**Table 1:** List of 5 data sets, the training and testing set of each data set for modelling

Data set	Training set	Testing set
1 (C4, C5&Cold station)	75% of observations randomly selected data set 1	The rest 25% of the observations
2 (C4, C5&Cold station)	The observations from C4 and C5	The observations from Cold station
3 (C4&C5)	75% of observations randomly selected from data set 3	The rest 25% of the observations
4 (C4&Cold station)	75% of observations randomly selected from data set 4	The rest 25% of the observations
5 (C5&Cold station)	75% of observations randomly selected from data set 5	The rest 25% of the observations

**Table 2:** Statistical parameters of partial least-squares (PLS), principal component (PCR) and multiple stepwise (MSR) regressions estimating DOC by spectral absorbance. Mean Bias error shows the difference between spectrophotometric measurements and DOC values measured by the thermal oxidation method (Multi N/C 2100). Training set are 75% (n=140) observations randomly selected from all samples; testing set are the rest 25% observations (n=43).

	Statistical Parameters	PLS	PCR	MSR
Training Set, n=140	r^2^	0.935	0.931	0.960
	RMSD (mg L^−1^)	3.310	3.406	2.611
	ncomp	6	6	7
Testing Set, n=43	r^2^	0.954	0.952	0.971
	RMSD (mg L^−1^)	2.877	2.953	2.352
	Mean Bias error (mg L^−1^)	0.086	0.070	-0.138

**Table 3:** Statistical parameters of partial least-squares (PLS), principal component (PCR) and multiple stepwise (MSR) regressions estimating DOC by spectral absorbance. Mean Bias error shows the difference between spectrophotometric measurements and DOC values measured by the thermal oxidation method (Multi N/C 2100). Training set are observations from C4 and C5 (n=150) while testing set are observations from Cold station (n=33).

	Statistical Parameters	PLS	PCR	MSR
Training Set,n=150	r^2^	0.905	0.873	0.926
	RMSD (mg L^−1^)	3.300	3.812	2.900
	ncomp	6	6	5

Testing Set, n=33	r^2^	0.694	0.712	0.704
	RMSD (mg L^−1^)	1.499	1.523	1.453
	Mean Bias error (mg L^−1^)	-5.793	6.220	-1.241

## 2 Experimental Design, Materials and Methods

### 2.1 Sampling and filtration

Before sample collection, the sampling bottles and reagent containers were cleaned in a Deko-2000 washer with detergent and soaked for at least 24 h in 2% HNO_3_, then rinsed six times with Milli-Q water. Glassware was additionally pre-combusted for 4 h at 450 °C before use.

In Cold station, water was sampled monthly in winter and fall, once a fortnight in spring, and every week in summer. In Krycklan, sampling was done monthly during winter, once a fortnight during summer and fall, and every third day during the spring flood. The water samples were filtered through Filtration Assembly with Whatman GF/F Glass Microfiber Filters (pore size 0.45 μm). To precondition the filtration system and avoid contamination from the filter, 30 ml of sample water was filtered and then discarded. As the sites locate in remote area, samples for absorbance measurements were preserved using ZnCl_2_ and then stored at 4 °C until laboratory analysis. Samples for DOC and Fe measurements were frozen until further analysis.

### 2.2 Laboratory measurements of spectral absorbance, DOC and Fe

After sample collection and preparation, spectral absorbance was measured with a laboratory benchtop spectrophotometer (UV-1800, Shimadzu, Kyoto, Japan) between 200 and 800 nm with a 10 mm pathlength quartz cell (acquisition step: 1 nm, scan speed: slow).

In Finland, dissolved organic carbon (DOC) was determined by thermal oxidation coupled with infrared detection (Multi N/C 2100, Analytik Jena, Germany) following acidification with phosphoric acid. Fe concentrations were determined calorimetrically with ferrozine corresponding to an absorbance at 562 nm by Victor3 1420 Multilabel Counter (PerkinElmer) [10]. In Sweden, DOC was measured with Shimadzu TOC-5000 using catalytic combustion [11]. Fe was analysed using Inductively Coupled Plasma Optical Emission Spectroscopy (ICP-OES Varian Vista Pro Ax) [12].

### 2.3 In-situ measurement of spectral absorbance and validation

In site, portable multi-parameter UV-Vis sensors (spectro::lyser, S::CAN Messtechnik GmbH, Austria) were applied as an emerging technology to monitor the water condition. UV-Vis sensors can determine the real-time spectral absorbance of water [5]. Thereafter, algorithms calculate DOC and Fe concentrations based on absorbance at a specific wavelength or multiple wavelengths. Three UV–Vis sensors were installed, one in the Yli-Nuortti river on June 12, 2018 and two in the Krycklan catchments on May 9, 2016. They measured absorbance across the UV–Vis range (220–732.5 nm, at 2.5 nm intervals) every 15 minutes and recorded these values in an internal datalogger. Water depth, temperature, turbidity and the voltage of S::CAN were detected simultaneously.

Unlike the laboratory benchtop spectrophotometer, in-situ S::CAN measured unfiltered water directly and was more sensitive to the environment changes such as water temperature, ambient sunlight and power supply. Therefore, the lab measured absorbance was used to check the performance of S::CAN and validate the quality of real-time spectral absorbance for DOC and Fe prediction (Fig. 3).

### 2.4 Modelling for DOC and Fe prediction

The real-time absorbance (every 15 mins) from S::CAN in C4,C5 and Cold station was integrated into daily data, then merged with lab measured DOC (n = 183) and Fe (n =142) according to date. The absorbance values from 220 nm to 732.5 nm at 2.5 nm intervals (207 variables) were used as input data for Fe analyses, while wavelengths shorter than 250 nm were excluded from the DOC analyses (194 variables) because inorganic substances can lead to interference at the lower end of the UV–Vis range [13].

We used three methods: multiple stepwise regression (MSR), partial least-squares regression (PLS), and principal component regression (PCR). These methods were selected due to their applicability to data sets containing collinear variables and datasets that may contain a larger number of independent variables than observations. Lab measured DOC and Fe concentrations were always the dependent variable, and the absorbance values at different wavelengths were the independent variables. The models rely on splitting the data into a training and testing data set. We tried 5 different splits of the data (Table 1). The performance of DOC prediction models (PLS, PCR, MSR) basing on data set 1 and 2 showed in Table 2 and Table 3, while the one (MSR) indicated in Table 4 basing on data set 3 to 5. Additionally, the performance of Fe prediction models (PLS, PCR, MSR) basing on data set 1 and 2 showed in Table 5 and Table 6, while the one (MSR) indicated in Table 7 basing on data set 3 to 5.

**Table 4:** The goodness of fit statistics of MSR regression estimating DOC by spectral absorbance for different data sets. Data set 3 is observations from C4 and C5; Data set 4 is observations from C4 and Cold station; Data set 5 is observations from C5 and Cold station. The training set contains 75% of observations that were randomly selected from each data set and the testing set contains the rest 25% of observations.

MSR	Statistical Parameters	Data set 3(C4&C5)	Data set 4(C4&Cold station)	Data set 5(C5&Cold station)
Training Set	r^2^	0.903	0.973	0.959
	RMSE (mg L^−1^)	3.243	2.599	1.787

Testing Set	r^2^	0.942	0.976	0.802
	RMSD (mg L^−1^)	2.797	2.424	4.177
	Bias (mg L^−1^)	-0.288	-0.203	1.369

**Table 5:** Statistical parameters of partial least-squares (PLS), principal component (PCR) and multiple stepwise (MSR) regressions estimating Fe ^3+^ by spectral absorbance. Training set are 75% (n=108) observations randomly selected from all samples; testing set are the rest 25% observations (n=34).

	Statistical Parameters	PLS	PCR	MSR
Training Set, n=108	r^2^	0.748	0.66	0.816
	RMSD (µg L^−1^)	433.592	503.991	370.633
	ncomp	8	8	7

Testing Set, n=32	r^2^	0.669	0.592	0.747
	RMSD (µg L^−1^)	570.403	637.597	502.047
	Mean Bias error (µg L^−1^)	-46.667	-79.032	-69.377

**Table 6:** Statistical parameters of partial least-squares (PLS), principal component (PCR) and multiple stepwise (MSR) regressions estimating Fe ^3+^ by spectral absorbance. Training set are observations (n=124) from C4 and C5, testing set are observations (n=18) from Cold station.

	Statistical Parameters	PLS	PCR	MSR
Training Set, n=124	r^2^	0.793	0.460	0.706
	RMSD (µg L^−1^)	168.933	196.038	139.652
	ncomp	10	10	9

Testing Set, n=16	r^2^	0.001	0.023	0.036
	RMSD (µg L^−1^)	251.723	238.010	240.025
	Mean Bias error (µg L^−1^)	-1461.512	-261.852	-13.408

**Table 7:** The goodness of fit statistics of the MSR regression estimating Fe by spectral absorbance for different data sets. Data set 3 is observations from C4 and C5; Data set 4 is observations from C4 and Cold station; Data set 5 is observations from C5 and Cold station. Training sets are 75% of the observations randomly selected from each data set and testing sets are the rest 25% of the observations.

MSR	Statistical Parameters	Data set 3(C4&C5)	Data set 4(C4&Cold station)	Data set 5(C5&Cold station)
Training Set	r^2^	0.868	0.9889	0.672
	RMSE (mg L^−1^)	287.398	108.905	473.997

Testing Set	r^2^	0.583	0.876	0.623
	RMSD (mg L^−1^)	619.901	378.814	479.334
	Bias (mg L^−1^)	179.009	-124.951	78.672

The ‘pls’ package [14] in R [15] was applied for PCR and PLS analyses. Coefficients and p-values were estimated by jackknife T-test method using ‘jack.test’ function in ‘pls’ package. MSR analyses were performed with ‘caret’ package [16] in R [15]. The correlation coefficient (R), root-mean-square deviation (RMSD), standard deviation (STD) and bias were used to check the performance of the models.

## CRediT Authors Statement

**Xudan Zhu:** Conceptualization, Methodology, Formal analysis, Writing - Original Draft, Visualization; **Liang Chen:** Methodology, Software, Writing- Reviewing and Editing; **Jukka Pumpanen:** Supervision, Writing - Reviewing and Editing; **Markku Keinänen:** Resources, Writing - Reviewing and Editing; **Hjalmar Laudon:** Data Curation, Writing - Reviewing andEditing; **Anne Ojala:** Writing - Reviewing and Editing; **Marjo Palviainen:** Writing - Reviewing andEditing; **Mikko Kiirikki:** Validation; **Kimmo Neitola:** Data Curation; **Frank Berninger:** Conceptualization, Supervision, Project administration, Funding acquisition, Writing - Reviewing and Editing.

## Declaration of Competing Interest

The authors declare that they have no known competing financial interests or personal relationships which have, or could be perceived to have, influenced the work reported in this article.

## Appendix Supplementary materials

Image, application 1
